# Maize growth response to different *Bacillus* strains isolated from a salt-marshland area under salinity stress

**DOI:** 10.1186/s12870-022-03702-w

**Published:** 2022-07-26

**Authors:** Maryam Zakavi, Hossein Askari, Mohammad Shahrooei

**Affiliations:** 1 Department of Plant Sciences and Biotechnology, Faculty of Life Sciences and Biotechnology, Shahid Beheshti University, Tehran, Iran; 2 Department of Microbiology and Immunology, Clinical and Diagnostic Immunology, KU Leuven, Leuven, Belgium; 3 Medical Laboratory of Dr. Shahrooei, Tehran, Iran

**Keywords:** Salt marshland, Salinity stress, *Bacillus*, Halotolerant, MALDI-TOF MS, Atomic Absorption Spectrometer (AAS)

## Abstract

Maize (*Zea mays*) growth performance has been hindered due to the high soil salinity. Salinity is one of the most severe abiotic stresses that has led to growth imbalance and profitability of harvests in arid and semi-arid regions. Plants have taken advantage of salt-tolerant bacteria as plant growth-promoters to enhance growth and reduce the adverse effects of salinity through the regulation of some biochemical, physiological, and molecular features. Preferences for non-chemical, eco-friendly, and economical approaches have caused the inquiry of the *Bacillus* genus as a joint group of plant growth-promoting rhizobacteria known to alleviate salt-stress impacts. In the present study, halotolerant *Bacillus* strains were isolated from salt-marshland soil and characterized for their physiological, molecular, and biochemical properties. Twenty-four bacterial isolates collected from high saline fields of salt marshland were analyzed by MALDI-TOF MS proteome analysis, which confirmed the taxonomic affiliation with *Bacillus cereus*, *Bacillus subtilis*, *Bacillus atrophaeus*, and *Bacillus thorngiensis*. Applying the isolates on maize plants as bio-inoculant bacteria obviously increased the growth parameters (*P* < 0.01). Pot experiments showed that isolates 74 and 90 were the most prominent strains to minimize the harmful effects of salinity. Its effects are heightening the potassium/sodium ratio and K-Na selectivity in shoots and roots measured by flame atomic absorption photometry (AAS). Accordingly, *Bacillus cereus* isolate 74 showed a maximum increase in dry weights of the shoot (133.89%), root (237.08%), length of the shoot (125%), and root (119.44%) compared to the control condition. Our findings suggest that bacteria isolated from marshland may be an economical and simple means to increase plant growth and resistance to high salinity soil conditions.

## Introduction

Soil salinization is a widespread threat to agriculture and rangeland productivity in Iran. Salt marshes are extremely biodiverse habitats with complex structures [27] which play pivotal roles in the eco-geomorphological evolution [51]. These areas provide critical ecosystems for various plants, animals, and microbes that are adapted to deal with full stress macro- and microenvironments [23]. Coevolution of plants and rhizobacteria under intensive stress may provide a new insight into discovering halophilic bacterial agents to cope with the adverse effects of salinity on plant growth parameters. It seems that some halophilic bacterial strains positively affect plant growth promotion by improving their salt removal efficiency [5].

The salt-tolerant plant growth-promoting rhizobacteria (PGPR) isolated from the saline condition can be used as probiotics for saline soil agriculture, which can be a promising substitute for improving crop yield approaches [3]. As frequently reported, halotolerant microbial isolates have greatly influenced the plant growth-promoting traits [36, 37]. Some *Arthrobacter*, *Bacillus*, and *Pseudomonas* species such as *Bacillus pumilus*, *B. aquimaris*, *B. arsenicus*, *B. sporothermodurances*, *Arthrobacter sp*., *B. cereus*, *B. subtilis*, and *P. medicona* from salt-affected rhizosphere significantly improved wheat growth by indole-3-acetic acid (IAA), siderophore and gibberellin production as well as phosphorus solubilization [47]. Beneficial effects of *Achromobacter piechaudii* ARV8 [36], *Stenotrophomonas rhizophila* strain DSM14405T [22], *Azospirillum* strains [13], *Bacillus cereus* [25], *Halobacillus* sp. and *Bacillus halodenitrificans* [8], *Pseudomonas syringae* and *Pseudomonas fluorescens* [31], *Zhihengliuella halotolerans*, *Staphylococcus succinus*, *Bacillus gibsonii*, *Oceanobacillus oncorhynchi*, *Halomonas* sp., and *Thalassobacillus* sp. [2], several salt-tolerant strains of *Rhodococcus*, *Oceanospirillales*, *Bacillales*, *Actinomycetales* [28]. *Leclercia adecarboxylata* [54] on different salt-affected plant species has been extensively investigated and confirmed. *Bacillus*, as a predominant bacterial genus under a stressed environment, is powered by an external shield of the formidable cell wall [18], endospore-forming ability [52], stable cell membrane [38], and internal enzymatic system [26] to cope and manage environmental hazards.

The present study aimed to isolate and characterize bacterial isolates collected from soil samples taken from one of the desert salt marshes in Iran. The potential of the bacterial isolates was assessed to promote maize growth parameters under salinity stress.

## Materials and methods

### Soil Samples

Soil samples were collected from a desert marshland located at the north eastern of Qom (longitude 51^o^14’13.92“ E and latitude 34^o^54’7.20” N). Eight soil samples were collected from 10 to 20 cm of the soil surface and completely mixed together and finally; one soil sample was transferred to the laboratory. The soil sample was dried in the dark and at room temperature for at least two days before sieving. Following the drying process, the soil sample was sieved through a 2 mm sieve to remove pebbles and other inert material and then kept in a zip lock cover.

### Bacterial Isolation

Eleven culture media, including Nutrient Agar (NA), Nutrient Agar plus MnSO4 (NA^+^ MnSO4), LB, Moller Hinton Agar (MHA), *Acidithiobacillus* (APH) medium, Violet Red Bile Lactose (VRB) agar medium, GYM Streptomyces medium, DPM medium, *Azospirillum* medium, and Azotobacter medium, were used. All media were sterilized at 121 °C for 20 min and plates were incubated inside polyethylene bags at 4 °C. For isolation of bacteria, 1 g of soil sample was suspended in 2 ml of sterile physiological saline (0.9% w/v NaCl) and then vortexed for 1 min. After sedimentation of soil particles, 100 ul of the supernatant was used for serial dilutions in the range of 10^−1^–10^−7^. Each dilution level was prepared in triplicate. Ten microliters of each dilution level were applied on the surface of solidified media by glass spreaders and incubated in an inverted position at 30 °C in the absence of light for 1–3 days. All instruments were sterilized completely before use and all dilution procedures were performed inside a laminar flow clean bench to achieve strict asepsis. Colonies were isolated based on the morphological characteristics (color, shape (top and side view), and diameter) using sterile toothpicks after two days of incubation at 30 °C. Accordingly, the number of each isolate was stated in colony-forming units per 1 g of soil (CFU g^−1^). Individual colonies were transferred and streaked separately on the same fresh media. Each plate was re-streaked twice to ensure strains purity and also cultured on the same liquid media for cryo-stock preparation. Long-term storage was carried out on a liquid medium containing 25% v/v glycerol at −70 °C.

### Gram stain, Oxidase, Catalase, and KOH testes

Gram staining of bacteria was examined after 48 h of incubation on MHA following the method of Bartholomew [6]. A non-staining KOH method [43] was performed to confirm the results of Gram staining. A catalase test was performed using 0.5 ml of 10% hydrogen peroxide solution and observed for the formation of gas bubbles. The oxidative activity of 95 isolates was studied with biochemical oxidase disks.

### Assessment of cold, dryness, salinity, heat, and pH on the bacterial growth

The growth of isolates (expressed as CFU) was evaluated in dark and under cold, drought, salinity, heat, and alkalinity stresses. Muller Hinton media was considered a basal growth media for all experiments. For cold and heat stresses, isolates were cultured at 15 and 60 °C for 10 hours, respectively. Basal media containing 100 mM NaCl, 25% PEG6000, and pH = 10 were prepared for salinity, drought, and alkalinity stresses and incubated for 10 h at 30 °C, respectively.

### MALDI-TOF MS profile acquisition

Isolates were sub-cultured twice on MHA at 30 °C for 24 h. Then approximately 100 μg of the bacterial colony were directly transferred to the MALDI target spot. Followed by drying at room temperature and overloading with 1uL of matrix solution (10 mg/ml a-cyano-4-hydroxycinnamic acid in 50% acetonitrile and 2.5% trifluoroacetic acid), each measurement was performed in two replications. MS analysis was carried out on an Autoflex MALDI-TOF mass spectrometer using Flex Control 3.4 software (Bruker Daltonics, Germany). Soil isolates with a valid MALDI-TOF MS score of 2 were undoubtedly assigned to the genus/species level.

### Bacterial identification

The bacterial classification was carried out using BioTyper 3.1 software (Bruker Daltonics, Germany). All identifications were reported with the following score values; unreliable identification was <1.7, 1.7–2.0 considered a possible genus identification and 2.0–2.3 expressed as a secure genus identification and probable species identification; and finally, >2.3 was regarded as highly probable species identification. Only the highest score value of all mass spectra belonging to individual cultures (biological and technical replicates) was recorded [40].

### Assessment of bacterial isolates on the maize growth

Maize seeds (*Zea mays*. Var Kosha) were obtained from Seed and Plant Improvement Institute of Karaj (Karaj, Iran, http://www.spii.ir/homepage.aspx?site=DouranPortal&tabid=1&lang=faIR), soaked in distilled water for 24 h and left to germinate. For the first irrigation, the sterilized MHA was treated with two salinity levels (0 and 100 mM NaCl) and bacterial isolates (CFU 2 × 10^3^) and used as irrigation water. Each treatment consisted of one bacterial isolate and one salinity level. After the first irrigation, a daily plant watering (5 ml/ per day) was carried out using sterilized pure water. A completely randomized block design with factorial treatments in three replications was employed for the experiment. After 20 days, plants were harvested, and shoot and root length (cm) and shoot and root fresh weight (mg) were successively recorded. For dry weight, samples were dried at 50 °C and measured after reaching a stable weight. Whole dry weight (mg), whole length (cm), and Shoot/Root were calculated. Sodium and potassium contents were extracted as described by Sakr et al., [32] and measured with an Atomic Absorption Spectrometer (AAS).

### Statistical analysis

All statistical analyses were performed by R software (version 3.6.2). The significance of the experiment was tested by one-way analysis of variance (ANOVA) and means separation was performed using Fisher’s protected Least Significant Difference (LSD) test at *P* < 0.01 by package Agricolae. Pearson correlation analysis was performed using SPSS Statistics for Windows, version 16.0 (SPSS Inc., Chicago, Ill., USA) to check the level of association between control/salinity stress and relative growth of soil isolates. Cluster analysis was done based on the Tomida report [46] using CLUSTER (version 3.0) software, and tree images were performed by Java Treeview (version 1.1.6r4). Hierarchical clustering was performed according to the Euclidian distance and complete linkage method.

## Results

### Isolation, characterization, and MALDI-TOF MS-based identification

Of 24 isolates, seven isolates were obtained on MHA, 2 on NA, 3 on NA^+^, 3 on LB, 2 on VRB, 3 on AZTO, and 4 on GYM through the morphological distinctions. All isolates presented a rode shape in cell form with mostly smooth and flat surface colony shape except for two isolates (35 and 120) by screening them on selective media. The isolates came in a variety of colors, including cream, white, yellow, orange, brick red, and red. Table 1 indicates soil isolates growth rates under selective media (e.i., MHA, NA, NA^+^, LB, VRB, AZTO, and GYM).

**Table 1:** Effect of selective, and MB media on microbial growth parameters, and morphological characterization of bacteria isolated from marshland

Isolate	Selective Microbial Media	Microbial Growth Parameters	Morphological characterization			
		CFU/ ml (*10^**5**^)	Color	Colony Size Score	Colony shape	
					Top view	Side view
33	MHA	9.0	Cream	3.0	Circular	Flat
34	MHA	8.5	Cream	5.0	Irregular	Flat
35	MHA	9.5	Cream	2.0	Circular	Raised
36	NA	4.5	Cream	10.0	Irregular	Flat
37	NA^+^	5.5	Cream	4.0	Circular	Flat
38	NA^+^	7.5	Cream	1.0	Circular	Flat
39	LB	7.0	Cream	3.0	Circular	Flat
73	VRB	4.0	White	1.0	Irregular	Flat
74	AZTO	1.0	Brick Red	4.0	Circular	Flat
89	GYM	9.0	White	10.0	Irregular	Flat
90	MHA	6.0	Cream	10.0	Irregular	Flat
91	MHA	4.5	Orange	5.0	Star shape	Flat
104	MHA	6.5	Cream	3.0	Circular	Flat
105	MHA	6.0	Red	1.0	Circular	Flat
106	NA	8.5	Cream	10.0	Irregular	Flat
120	NA^+^	7.0	Cream	8.0	Circular	Raised
121	LB	8.0	Cream	10.0	Circular	Flat
128	LB	7.5	Yellow	7.0	Circular	Flat
129	VRB	4.5	White	1.0	Circular	Flat
130	AZTO	1.5	Brick Red	5.0	Circular	Flat
146	AZTO	2.2	White	3.0	Circular	Flat
147	GYM	5.0	White	10.0	Irregular	Flat
148	GYM	7.5	Yellow	3.0	Circular	Flat
149	GYM	7.0	White	10.0	Irregular	Raised

Table 2 shows the effect of abiotic stresses on bacterial growth. Accordingly, under salinity stress, isolates 36, 105, 106, 120, 121, and 128 showed growth equal to or greater than normal conditions. Although isolates 33, 35, 39, 73, 74, 90, 129, 130, and 149 also had significant growth under salinity stress, their growth retardation was minimal under salinity stress. In cold stress, only isolate 36 could grow higher than the control, and the rest of the isolates had reduced growth. Under heat stress, the growth of isolate 146 was 1.3 times higher than the control under stress conditions, but the rest of the samples had a considerable growth reduction. Under heat stress, the development of 146 isolates was 1.3 times higher than the control under stress conditions, but the rest of the samples had a large growth reduction. At a higher pH level (pH = 10), isolates 89 and 146 showed better growth under stress than normal conditions, and the development of the rest isolates was decreased; however, isolates 90, 147, and 148 had acceptable change compared to controls. Also, all isolates under drought stress had severe growth retardation.

**Table 2:** Effect of cold, dryness, and salinity stresses on the growth of isolates compared to control conditions after 10 h in bacteria isolated from marshland

Isolate	Normal Condition	Salt Stress	Drought Stress	Cold Stress	Heat Stress	pH
	CFU/ml (*10^**5**^)	CFU/ml (*10^**5**^)	CFU/ml (*10^**5**^)	CFU/ml (*10^**5**^)	CFU/ml (*10^**5**^)	CFU/ml (*10^**5**^)
33	9.0	4.94	1.89	4.32	1.23	1.05
34	8.5	3.86	2.49	4.98	2.52	2.06
35	9.5	6.77	2.50	5.67	2.21	2.56
36	4.2	4.23	1.34	4.33	1.68	1.93
37	11.2	8.80	3.72	5.16	2.14	2.59
38	6.0	4.50	2.52	3.13	2.89	3.86
39	6.0	5.71	2.10	4.76	2.41	3.05
73	5.6	5.66	1.64	3.71	1.89	3.24
74	6.2	5.91	1.69	4.71	1.30	1.98
89	6.0	4.72	2.13	2.30	3.13	6.82
90	6.0	4.07	1.48	4.29	4.07	5.16
91	4.5	1.90	1.26	3.27	0.90	0.99
104	6.5	1.95	1.68	1.91	0.78	0.94
105	6.0	4.97	1.45	1.94	1.17	1.85
106	4.0	4.20	1.37	3.07	1.28	2.29
120	5.0	5.11	1.78	2.55	1.56	2.41
121	4.8	4.82	1.22	2.06	3.56	2.80
128	5.4	5.49	1.44	3.71	3.62	3.04
129	4.6	4.50	1.41	3.43	0.95	1.39
130	4.4	4.24	1.18	1.45	3.90	2.85
146	4.0	2.31	0.83	1.05	5.41	4.82
147	7.0	6.17	3.16	2.93	3.59	6.33
148	7.2	6.03	2.25	3.14	3.93	6.48
149	5.8	5.10	1.99	2.87	2.36	2.95

Molecular characterization and biochemical analysis were done on bacterial isolates with catalase, KOH degradation, and oxidase abilities. All 24 isolates were shown Gram-positive and minus catalase reactions. However, there were differences between the isolates for oxidase and KOH degradation tests. In this regard, only four isolates showed KOH digestion activity (33, 36, 104, and 130) and six isolates did not show oxidase activity (37, 90, 121, 128, 130, and 147). Table 3 summarizes the results of the identification of isolates by the MALDI-TOF method and the results revealed a close relatedness of the isolates to the *Bacillus* genus. Protem analysis of the isolates produced an exact match to four species during MALDI-TOF and the results showed all the isolates belonged to *B.cereus*, *B atrophaeus*, *B.subtilis*, and *B.thuringiensis*. Besides, the results of biochemical tests and Gram staining confirmed the results of MALDI-TOF. In this way, all isolates were circular, Gram-positive, and produced endospores; so, they were also characterized based on distinguishing features.

**Table 3:** Taxonomic assignment of salt marshland isolates by MALDI-TOF and biochemical tests

Isolate	Bacterial name	NCBI Identifier	MALDI-TOF Score	Biochemical tests			
				Gram Stain	Catalase	KOH	Oxidase
33	*B. cereus*	1396	2.15	+	−	+	+
34	*B. cereus*	1396	2.08	+	−	−	+
35	*B. cereus*	1396	2.25	+	−	−	+
36	*B. atrophaeus*	1452	2.45	+	−	+	+
37	*B. subtilis*	1423	2.03	+	−	−	−
38	*B. thuringiensis*	1428	2.13	+	−	−	+
39	*B. cereus*	1396	2.23	+	−	−	+
73	*B. subtilis*	1423	2.04	+	−	−	+
74	*B. cereus*	1396	2.26	+	−	−	+
89	*B. cereus*	1396	2.18	+	−	−	+
90	*B. atrophaeus*	1452	2.07	+	−	−	−
91	*B. cereus*	1396	2.15	+	−	−	+
104	*B. cereus*	1396	2.03	+	−	+	+
105	*B. cereus*	1396	2.07	+	−	−	+
106	*B. cereus*	1396	2.14	+	−	−	+
120	*B. thuringiensis*	1428	2.26	+	−	−	+
121	*B. cereus*	1396	2.11	+	−	−	−
128	*B. cereus*	1396	2.14	+	−	−	−
129	*B. cereus*	1396	2.12	+	−	−	+
130	*B. cereus*	1396	2.09	+	−	+	−
146	*B. cereus*	1396	2.03	+	−	−	+
147	*B. cereus*	1396	2.25	+	−	−	−
148	*B. cereus*	1396	2.21	+	−	−	+
149	*B. cereus*	1396	2.11	+	−	−	+

### Effect of isolated bacterial strain on Maize plant growth parameters under control conditions and salinity stress

The results of the application of isolates on plants indicated that yield per plant and growth parameters were significantly boosted by bacterial inoculation under salinity stress conditions; so isolates could alleviate the deleterious impacts of salinity on the growth of maize plants under natural salinity field conditions. Shoot dry weight, root dry weight, shoot length, and root length readings in maize leaves and roots were significantly increased by bacterial isolates under salinity stress. Hence, bacterial assessments improved the growth parameters of maize plants compared with the non-inoculated control also under salinity stress.

The results of tests under salinity conditions showed distinct differences in the shoot and root growth between isolates’ treatments. Plant-microbe interactions under salinity stress were statistically significant (*p* < 0.001). Four growth parameters were associated with salt tolerance analysis in maize and the analyses were linked to shoot dry weight, root dry weight, shoot length, and root length of maize plants under normal and salinity conditions. Table 4 demonstrated the effect of soil isolates on maize plants’ growth under normal conditions and salinity stress. As the results showed, isolates 33, 74, 90, and 130 obviously have boosted dry weight under stress conditions compared to the control, whereby a strong increase in root length and weight were recorded. Also, isolates 33, 74, 90, 105, 130, and 128 showed better effects on maize root dry weight than the control under salinity stress. Besides, 128 bacteria had the same impacts on root dry weight under both salinity and normal conditions. In general, isolates 73, 74, 90, 105, and 130 resulted in a higher total weight of maize plants under salinity stress than normal conditions. In addition to maintaining plant growth, the treatment with bacteria-initiated plant growth in salinity stress compared to the control.

**Table 4:** Influence of soil isolates and salinity conditions on growth parameters after 20 days of assessment on maize plants

Isolate	Bacterial name	Shoot DW (cm)		Root DW (cm)		Shoot length		Root length	
		Salinity	Control	Salinity	Control	Salinity	Control	Salinity	Control
33	*B. cereus*	93.25	120.75	170.75	243.25	24.25	28.50	4.75	4.50
34	*B. cereus*	73.25	93.25	140.75	178.25	19.00	24.50	3.63	6.00
35	*B. cereus*	53.25	80.75	160.75	305.75	12.75	25.00	2.75	4.75
36	*B. atrophaeus*	65.75	123.25	180.75	313.25	14.75	25.50	4.00	4.75
37	*B. subtilis*	63.25	120.75	160.75	273.25	16.75	29.25	3.63	4.75
38	*B. thuringiensis*	60.75	123.25	160.75	448.25	15.00	27.25	4.25	7.00
39	*B. cereus*	35.75	88.25	108.25	98.25	9.50	21.00	2.00	3.75
73	*B. subtilis*	105.75	135.75	285.75	215.75	21.25	25.50	3.75	6.00
74	*B. cereus*	138.25	103.25	410.75	173.25	28.75	23.00	5.38	4.50
89	*B. cereus*	83.25	85.75	173.25	245.25	19.00	26.50	4.00	5.25
90	*B. atrophaeus*	103.25	98.25	290.75	228.25	18.50	27.50	4.00	5.63
91	*B. cereus*	85.75	135.75	208.25	235.75	19.75	27.75	3.75	5.75
104	*B. cereus*	45.75	85.75	185.75	235.75	14.00	22.25	3.00	5.00
105	*B. cereus*	43.25	68.25	285.75	198.25	13.50	20.00	2.75	3.50
106	*B. cereus*	58.25	103.25	200.75	193.25	13.50	23.25	3.75	5.25
120	*B. thuringiensis*	65.75	108.25	150.75	210.75	20.25	29.75	3.25	4.50
121	*B. cereus*	65.75	120.75	130.75	220.75	15.75	26.00	4.75	5.50
128	*B. cereus*	60.75	110.75	160.75	148.25	14.25	26.50	3.38	6.00
129	*B. cereus*	60.75	65.75	130.75	130.75	17.25	20.75	3.38	3.88
130	*B. cereus*	88.25	78.25	193.25	160.75	24.50	20.50	4.25	4.00
146	*B. cereus*	73.25	85.75	135.75	203.25	19.75	23.50	4.50	4.25
147	*B. cereus*	50.75	80.75	155.75	138.25	14.50	21.75	4.00	4.00
148	*B. cereus*	45.75	75.75	110.75	178.25	13.75	23.75	3.50	3.13
149	*B. cereus*	55.75	88.25	203.25	330.75	10.50	24.25	2.88	4.50
150	*Control +*	73.25	103.25	155.75	170.75	19.50	30.75	4.13	6.13
152	*Control* -	0.00	88.25	0.00	208.25	0.00	32.25	0.00	9.00
*LSD Value*		0.04026911		0.4855884		13.33303		9.250745	

**Fig. 1:**
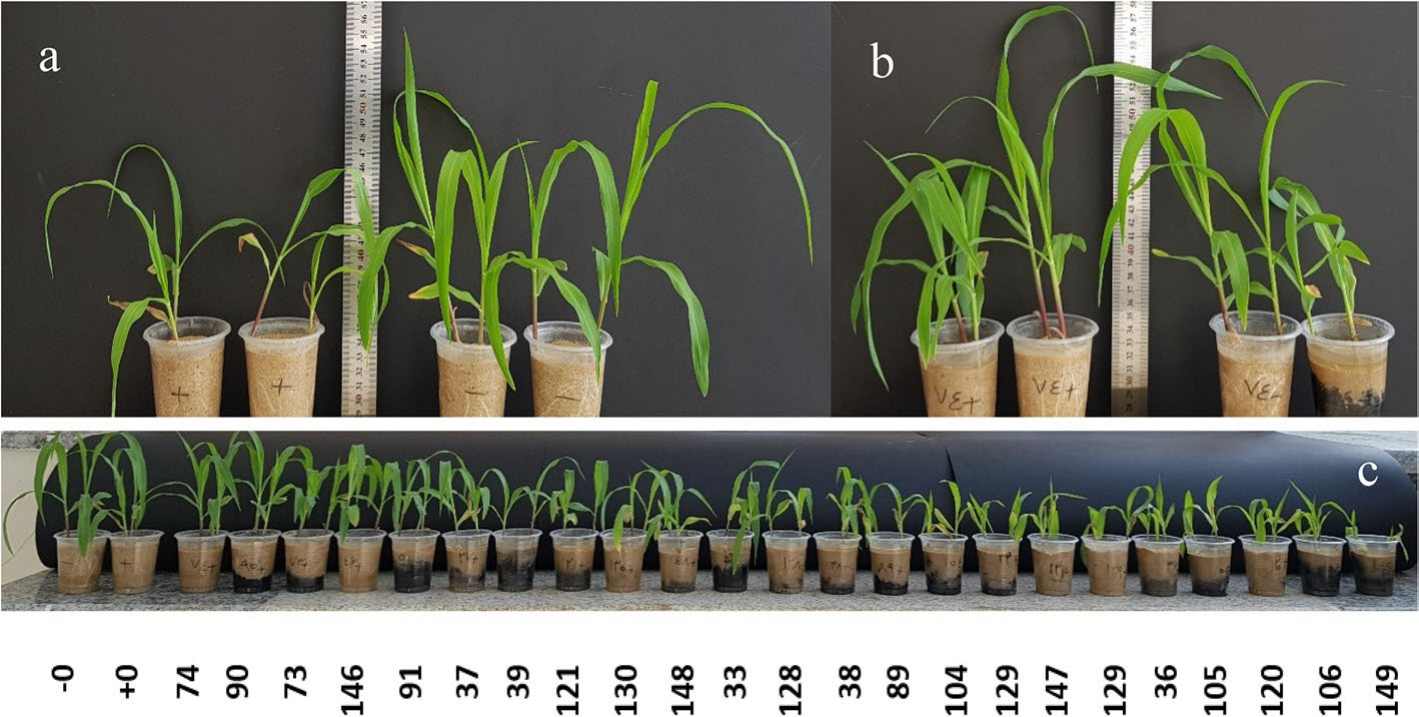
Effects of bacterial inoculation on the growth of *Zea mays* seedlings stressed with NaCl. **a** Aerial view and whole seedlings of control (non-inoculated) plants and (**b**) those inoculated with strain 74 exposed to 100 mM NaCl stress. “–” and “+” indicate uninoculated controls under normal (0-) and salinity stress (0+) conditions, respectively. **c** Total controls plants and inoculated plant under salinity stress‘

Isolates 74 and 130 possessed a higher shoot length under stress than control conditions (Fig. 1a,b) and isolates of 33, 74, 130, and 146 had the same impact on the length of root in maize plants. Moreover, isolates 90 and 130 also showed the highest effect on plant length (shoot + roots) under stress conditions compared to the control. Isolate 105 also was more effective in shoot length, and this could be due to the induction of auxin production by *Bacillus* isolate 105.

In contrast, isolates 35, 38, 39, 104, 105, 120, 121, 128, 129, 147, 148, and 149 caused weak shoot dry weight in comparison to +control under salinity stress. All isolates had higher impacts on root dry weight than the +control. Further, 36, 37, 38, 39, 104, 105, 106, 128, 147, 148 and 149 resulted in lower shoot length and inhibition of shoot formation compared to +control. Isolate 39 had an adverse effect on root length than +control (Fig. 1c).

### The Na^+^ and K^+^ levels in shoot and root in both salinity and normal conditions

The highest values for total plant biomass were found in the plants inoculated with isolates 74 and 90 under both non-saline conditions and NaCl stress (Table 5). According to the results, the Na^+^ and K^+^ contents in the shoots and roots were affected by bacterial application under both saline and non-saline conditions, with a significant reduction in Na^+^ levels in the leaves and roots compared to the un-inoculated control. At 100 mM NaCl, theresults of LSD analysis of Na and K contents in shoots and roots of maize plants were shown a significant difference between control and salt stress conditions at the level of *P* < 0.001. Table 5 represents the amounts of sodium and potassium ions in the root and leaf tissue of maize under normal and salinity stress. As shown, the amount of Na was at the lowest level in maize plants at 100 mmol/l NaCl salinity condition, which was inoculated with isolate 74; so isolate 74 led to less salt absorbance. Also, the foliar and fibroid levels of K increased with the inoculation of this bacterial isolate.

**Table 5:** Effect of inoculation with isolates under control and salinity stress on the amount of Na and K in shoot and root tissues of maize plants measured by AAS

Isolate	Bacterial Name	Shoot						Root					
		Na (mg/g DW)		K (mg/g DW)		Na/K		Na		K		Na/K	
		Control	Salinity	Control	Salinity	control	Salinity	Control	Salinity	Control	Salinity	control	Salinity
33	*B. cereus*	47.86	68.63	57.78	43.98	0.84	1.57	48.59	68.01	19.73	25.04	2.51	2.74
34	*B. cereus*	58.68	66.40	63.30	27.57	0.93	2.51	58.76	65.00	30.06	24.55	1.97	2.65
35	*B. cereus*	60.04	65.02	61.70	24.84	0.98	2.78	61.18	57.20	19.69	17.56	3.13	3.26
36	*B. atrophaeus*	55.43	68.70	57.95	32.25	0.96	2.13	66.95	68.70	23.05	12.32	2.92	5.69
37	*B. subtilis*	40.54	68.70	32.46	26.93	1.64	2.58	67.94	65.91	23.68	13.20	2.87	5.25
38	*B. thuringiensis*	71.92	87.03	77.30	37.90	0.93	2.30	65.18	61.03	34.80	17.81	1.93	3.43
39	*B. cereus*	61.71	64.54	55.75	20.98	1.20	3.51	67.82	60.77	38.31	15.83	1.77	3.84
73	*B. subtilis*	81.56	87.20	64.95	55.82	1.26	1.56	80.94	75.48	36.19	21.95	2.24	3.43
74	*B. cereus*	60.20	59.02	66.22	61.92	0.91	0.96	32.54	72.91	31.18	19.05	1.04	4.55
89	*B. cereus*	49.86	56.79	46.78	28.36	1.07	2.04	63.71	49.95	23.68	6.36	2.69	7.89
90	*B. atrophaeus*	25.76	35.19	43.32	11.68	0.60	3.32	40.37	58.55	11.33	18.00	3.63	3.25
91	*B. cereus*	1.82	5.24	83.78	53.22	0.02	0.10	12.93	22.31	52.45	13.95	0.24	1.61
104	*B. cereus*	70.46	82.32	35.33	28.19	2.02	2.92	65.74	68.79	13.06	15.34	6.98	4.49
105	*B. cereus*	70.24	78.75	6.44	20.66	11.25	3.81	61.45	80.07	13.54	20.14	4.55	4.02
106	*B. cereus*	54.92	69.48	66.75	39.68	0.82	1.75	68.77	75.71	26.54	28.65	2.59	2.80
120	*B. thuringiensis*	51.66	64.76	69.90	48.11	0.75	1.35	49.50	37.26	31.24	20.43	1.65	1.94
121	*B. cereus*	82.46	98.26	68.85	46.19	1.20	2.14	80.55	71.27	34.25	23.07	2.36	3.12
128	*B. cereus*	86.70	87.02	64.54	36.64	1.36	2.38	76.69	78.04	25.11	16.90	3.04	4.65
129	*B. cereus*	65.72	78.55	37.25	33.99	1.76	2.34	58.39	71.69	13.24	18.49	4.46	3.89
130	*B. cereus*	73.21	70.04	57.10	37.37	1.30	1.88	71.22	84.00	23.47	27.32	3.13	3.08
146	*B. cereus*	49.63	85.32	68.45	23.03	0.73	3.71	62.45	85.64	26.51	28.99	2.39	2.98
147	*B. cereus*	53.30	78.40	57.89	37.38	0.92	2.13	46.57	70.55	24.99	18.55	1.89	4.33
148	*B. cereus*	53.81	78.52	52.76	27.77	1.02	2.85	53.97	69.79	25.84	17.46	2.12	4.33
149	*B. cereus*	51.09	87.01	47.11	25.51	1.09	3.41	55.91	78.71	22.46	17.22	2.51	4.59
Control	*0*	20.06	20.06	40.32	40.32	0.49	0.49	54.55	54.55	18.71	18.71	2.96	2.96
	*minus/plus*	62.78	76.39	62.11	39.06	1.01	1.99	63.00	74.40	34.32	13.62	1.84	6.00
*LSD Value*		13.26		16.37		1.13		14.34		10.20		2.17	

### Pearson correlation

Research efforts investigating the effects of salt-marsh isolates on plants grown under salinity stress have led to the characterization of different microbial behaviors under stress conditions, which involve their roles in creating salinity tolerance in maize plants. The Pearson correlation coefficient between plant growth parameters under control and salinity stress and bacterial growth in the control (Table 6) and stresses (Table 7) conditions reflects a significant positive correlation between shoot dry weight and other plant growth parameters under control and salinity stress (Tables 6 and 7). Furthermore, bacteria growth under salt stress had a great correlation with its growth under drought and cold stress (Tables 6 and 7) as well bacterial growth under heat and pH stresses (Tables 6 and 7) under both salinity and control conditions.

**Table 6:** Pearson correlation coefficients (r) between maize plants’ growth parameters in the control conditions and the growth of bacteria under stresses conditions

Plant growth parameters							Bacterial growth					
Control condition	Shoot DW (mg)	Root DW (mg)	Whole weight (mg/plant)	Shoot length (cm)	Root length (cm)	Whole length (cm)	Normal condition	Salt stress	Drought stress	Cold stress	Heat stress	pH
**Shoot DW (mg)**	1											
**Root DW (mg)**	0.391*	1										
**Whole weight (mg/plant)**	0.587**	0.975**	1									
**Shoot length**	0.563**	0.346 ns	0.441*	1								
**Root length**	0.418*	0.301 ns	0.366 ns	0.689**	1							
**Whole length**	0.559**	0.356 ns	0.449*	0.978**	0.826**	1						
**Normal condition**	0.066 ns	0.135 ns	0.135 ns	0.328 ns	−0.026 ns	0.264 ns	`1					
**Salt stress**	−0.001 ns	−0.012 ns	−0.011 ns	0.140 ns	−0.224 ns	0.055 ns	0.813**	1				
**Drought stress**	−0.018 ns	0.210 ns	0.180 ns	0.216 ns	0.001 ns	0.178 ns	0.885**	0.684**	1			
**Cold stress**	0.263 ns	0.077 ns	0.131 ns	0.289 ns	0.166 ns	0.284 ns	0.581**	0.524**	0.491*	1		
**Heat stress**	−0.196 ns	−0.084 ns	−0.122 ns	−0.006 ns	−0.005 ns	−0.007 ns	0.144 ns	0.029 ns	−0.014 ns	−0.287 ns	1	
**pH**	−0.273 ns	−0.046 ns	−0.107 ns	0.006 ns	−0.096 ns	−0.021 ns	0.297 ns	0.208 ns	0.265 ns	−0.208 ns	0.754**	1

**r: Significantly different at *P* < 0.01; *r: Significantly different at *P* < 0.05; *ns* Not significantly different (*P* > 0.05)

**Table 7:** Pearson correlation coefficients (r) between maize plants’ growth parameters under salinity stress and growth of bacteria under stresses conditions

Plant growth parameters							Bacterial growth					
Salinity stress	Shoot DW (mg)	Root DW (mg)	Whole weight (mg/plant)	Shoot length (cm)	Root length (cm)	Whole length (cm)	Normal condition	Salt Stress	Drought Stress	Cold Stress	Heat stress	pH
**Shoot DW (mg)**	1											
**Root DW (mg)**	0.688**	1										
**Whole weight (mg/plant)**	0.817**	0.981**	1									
**Shoot length**	0.879**	0.471*	0.611**	1								
**Root length**	0.717**	0.309 ns	0.439*	0.739**	1							
**Whole length**	0.884**	0.463*	0.607**	0.995**	0.802**	1						
**Normal condition**	−0.028 ns	−0.111 ns	−0.096 ns	−0.069 ns	−0.051 ns	−0.068 ns	1					
**Salt stress**	−0.076 ns	0.005 ns	−0.017 ns	−0.120 ns	−0.122 ns	−0.125 ns	0.813**	1				
**Drought stress**	−0.250 ns	−0.221 ns	−0.243 ns	−0.230 ns	−0.188 ns	−0.232 ns	0.885**	0.684**	1			
**Cold stress**	0.159 ns	0.108 ns	0.129 ns	−0.025 ns	−0.131 ns	−0.041 ns	0.581**	0.524**	0.491*	1		
**Heat stress**	−0.017 ns	−0.289 ns	−0.233 ns	−0.039 ns	0.233 ns	−0.001 ns	0.144 ns	0.029 ns	−0.014 ns	−0.287 ns	1	
**pH**	−0.055 ns	−0.170 ns	−0.150 ns	−0.136 ns	0.112 ns	−0.104 ns	0.297 ns	0.208 ns	0.265 ns	−0.208 ns	0.754**	1

**r: Significantly different at *P* < 0.01; *r: Significantly different at *P* < 0.05; *ns* Not significantly different (*P* > 0.05)

### Cluster analysis of plant responses to salinity under inoculation of 24 soil isolates

The abiotic stress caused by enhancing chemicals such as Na is a common phenomenon occurring in the rhizosphere. It is responsible for the presence of a wide variety of physiological processes in plants. The application of the salt-tolerant bacteria, which naturally reside in the rhizosphere as plant growth promoters, provides a natural way to boost plant species’ growth and resistance.

**Fig. 2:**
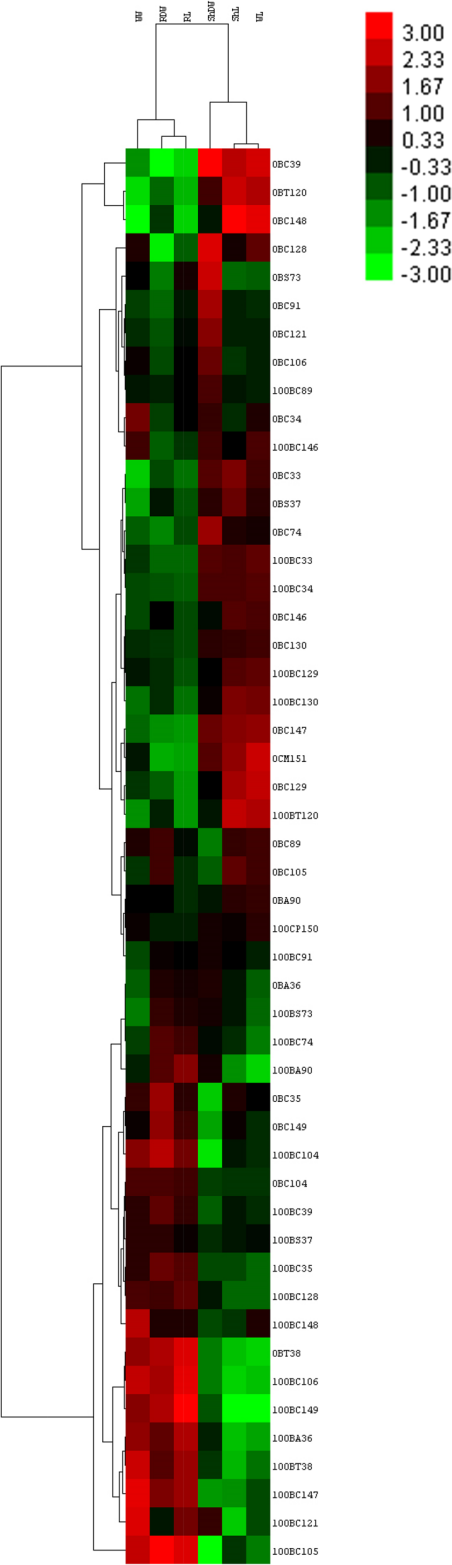
Represents Hierarchical cluster analysis of the effect of isolates on maize plants under control and salinity stress (100 mM salt stress) drown by CLUSTER and Treeview softwares. Hierarchical clustering was done based on the Euclidian distance and complete linkage method. Colors indicate the impacts of isolates on the plants. Accordingly, red, green, and black colors show positive, negative, and no-effect isolates, respectively. The horizontal axis indicate plant growth parameters: Whole weight (WW), Root dry weight (RDW), Root length (RL), Shoot dry weight (ShDW), Shoot length (ShL), Whole length (WL). Vertical axis showes the assayed bacterial isolates

The results of clustering of plant growth under salinity stress in the presence and absence of isolates showed that, in terms of affinity, the growth parameters WW, RDW, and RL come together in one group, and ShW, ShL, and WL in another group (Fig. 2). Isolates are also classified into two main groups in terms of their effect on plant growth. The first group showed better effectiveness on ShL, WL, and ShW, and the second group was more effective on RW, WW, and RL (Fig. 2).

## Discussion

Several abiotic elements may play a key role in modulating the diversity of soil microbes, including those inhabiting the rhizosphere [34], and one of the most important abiotic stresses is high-salt concentration. According to the previous reports, approximately 831 million hectares of the land area are affected by salt worldwide [39]. Salinity impedes photosynthesis and increases photorespiration, thereby altering the normal ion homeostasis of cells by providing nutrient imbalance, which is caused by loss of the plant’s ability to control nutrient uptake and/or transport from root to shoot, leading to ion deficiencies [35]. Microbial forms of life can be found over a vast range of salt concentrations. Furthermore, these organisms cope with hyperosmotic stress by utilizing various strategies [41] to mitigate salinity and boost the efficiency of plants [9]. Among bacteria, the genera *bacilli* and *pseudomonas* are two of the most critical and common plant growth promoters [9, 19, 21, 24, 30]. Also, it has been demonstrated that the existence of indigenous marshland isolates is still important to study [42], as it will open an opportunity to develop local-strain-based plant promoters’ productions against abiotic stresses such as salinity which is very common in Iran.

Several reports have indicated that the sequences of dominant *Bacillus* species present in different soils are not the same as those present in easily cultured isolates [55]. It was demonstrated that not only *Bacilli* isolates could enhance the accumulation of amino acids and carbohydrates but also reduce the antioxidant activity of enzymes such as catalase and glutathione peroxidase and electrolyte leakage in *Zea mays* [48].

Root-associated microorganisms play a critical role in retaining soil humidity and promoting plant growth under abiotic stresses by available services like microbial ecological services, protection from the soil mechanical stress, protection from osmotic and oxidative stresses, and effects on hormone homeostasis [11].

The findings of the present study revealed that shoot, root dry weight per plant, and length of shoot and root were significantly increased by bacterial applications under salinity stress conditions. Inoculation of isolates significantly (*P* < 0.01) increased shoot dry weight and root dry weight, which are useful measures of the physiological stability of plants. The exploitation of soil microbes for utilizing salt-stressed land is a useful method that may provide a quick-fix solution to salinity [44] as marshland microbes show different types of metabolic and adaptive responses to the variable supply of water, oxygen, organic/inorganic substrates and other available nutrients [4, 15]. The effect of isolates on the growth parameters of maize under salinity stress proved that bacteria were divided into three groups in terms of effectiveness: a) Isolates with a favorable effect on plant growth, b) Isolates with adverse effects on plant growth, and c) Isolates with no impact on plant growth. Hence it has been proved that bacteria of the *Bacillus* genus have been widely reported as promising candidates for bacterization because of their ability to eliminate or alleviate the harmful effects of saline stress, regulate plant physiological characteristics, and promote plant growth [14, 19, 29, 49, 53, 56]. Also, Wang et al. [50] reported increased shoot length of capsicum at 300 mM salt concentration when treated with *B. megaterium.*

In summary, the findings of the current study suggest that bacterial isolates belonging to *Bacillus* are dominant in the rhizosphere of salt marshland. Under salinity stress, 29% of isolates had a significant effect on shoot weight, 50% on root weight, 16% on shoot length, and 8% on root length. It has been established those high concentrations of sodium and chloride in soil may depress nutrient ion activities and produce extreme ratios of Na/Ca and Na/K in the plants causing the plants to be susceptible to osmotic and specific ion injury, as well as to nutritional disorders, resulting in reduced yield and quality [16, 45]. Based on our findings, the observed increase in the maize growth variety on inoculation with *Bacillus sp* under salt stress has been supported by reducing the Na/K ratio. Hence our results showed isolates 74, 33, and 91 reduced this ratio and increased salt tolerance in maize plants. Potassium (K) plays a crucial role in water stress tolerance in salinity stress. It is an osmotically active compound that contributes to water absorption in the root [17, 20]. AbdElgawad et al. reported that maize plants could increase enzymatic and nonenzymatic antioxidants under salt stress to prevent harmful effects on salinity [1]. Also, it was shown that increase in phosphatidylglycerol, as well as a decrease in phosphatidylethanolamine and linoleic acid, correlated with salt tolerance in *Zea mays* [33]. Accordingly, we conclude that bacterial isolates are involved in salinity tolerance in a way to aids the plant in boosting such antioxidants and lipids.

Indeed, salt marsh sediment bacteria remain primarily in a black box regarding their diversity and functional roles through salt marsh benthic food web pathways [12]. Therefore, we investigated whether it is possible to isolate aerobic bacteria with efficacy for salt tolerance from salt-marshland and whether these bacteria can also induce salinity tolerance in maize plants. We conducted this study on a salt marsh because the spatial ecology of salt marshes is exceptionally well understood; so bacteria exhibited in salt marshes are important in many ways. For instance, bacterial isolates from the rhizosphere of salt marsh show host specificities on plant vegetation in terms of composition [7] as well as in terms of abundance and heterotrophic activity [7, 10]; thus reflecting the adaptation to distinct environmental pressures. These shreds of evidence were confirmed by the data in the present study showing that salt-marshes are profitable and dynamic ecosystems with chemical and physical gradients that lead to discovering aerobic bacteria with salt tolerance efficacy, which can mitigate deleterious effects of salt stress and also induce salinity tolerance in plants.

## Conclusion

Assessing *Z. mays* plants with beneficial soil bacterial isolates have a great impact on plant growth and their persistence over abiotic stresses such as salinity. It is important to find practical approaches to isolate and characterize such bacteria. One of the most critical steps is how to figure out the correct place to isolate these bacteria. The particular conditions of salt marshlands can provide a unique environment for the emergence of salt-resistant bacteria. Many of these kinds of isolates, as well as resisting salt stress, may create resistance in surrounding organisms. The main challenge in this study was to survey the effectiveness of isolates on plant growth and their salt resistance. Our findings indicate that *B. subtilis* 73, *B. cereus* 74, and *B. atrophaeus* 90 are talented salt-marshland strains that need to be more analyzed in further studies for their application feasibility on the maize crop.

## Data Availability

The datasets used and/or analyzed during the current study available from the corresponding author on reasonable request.
